# Perception and satisfaction of blind individuals regarding an electronic cane prototype with a wearable haptic device

**DOI:** 10.5935/0004-2749.2023-0317

**Published:** 2024-09-16

**Authors:** Caio Henrique Marques Texeira, Ana Luiza Fontes de Azevedo Costa, Amanda Amorim Rodrigues, Vagner Rogério dos Santos, Adriana Berezovsky

**Affiliations:** 1 Departamento de Oftalmologia e Ciências Visuais, Escola Paulista de Medicina, Universidade Federal de São Paulo, São Paulo, SP, Brazil

**Keywords:** Blindness, Canes, Patient satisfaction, Perception, Haptic technology, Wearable electronic devices, Surveys and questionnaires

## Abstract

**Purpose:**

This study aimed to evaluate the perception and degree of satisfaction of blind individuals regarding an electronic cane prototype with a wearable haptic interface.

**Methods:**

Two scenarios with different obstacles were created to conduct tests with the canes (the user’s cane and the prototype one). The perception and satisfaction of participants regarding the electronic cane were assessed using a questionnaire, the number of collisions during the tests, and the time each individual took to complete the course in each scenario.

**Results:**

Ten blind individuals who used the white cane participated in this study. Eight were males, and two were females. Their age ranged from 23 to 43 (average 32.3 ± 7.13 years and median 32 years). There was a tendency for fewer collisions with ground obstacles when the electronic cane was used than when the white cane was used. However, there was no statistically significant difference between the number of collisions and the course completion time in each scenario with either canes tested.

**Conclusion:**

Overall, the perception and satisfaction of individuals regarding the prototype used were positive.

## INTRODUCTION

Recent data from the Vision Loss Expert Group estimated that there are 553 million individuals with visual impairment worldwide. Among these, 43 million are legally blind, representing 0.49% of the world’s population. In Brazil, 18 million are visually impaired and 1.7 million are blind. Visual impairment is defined as the best corrected visual acuity of <20/40 (0.3 logMAR) to 20/400 (1.3 logMAR) in the best eye. Blindness is the best corrected visual acuity of <20/400 (1.3 logMAR) and/or visual field < 10° in the best eye^(1^,^2)^.

The sense of vision is very important for everyday tasks. Visual impairment or loss imposes difficulties or even impossibilities in performing some tasks. One task affected by visual impairment is mobility, which is the ability to safely, comfortably, and efficiently navigate the environment using the remaining senses. The remaining senses of hearing, touch and smell, the vestibular system and the muscular memory assist in perceiving nonvisual stimuli^(3^,^4^,^5^,^6^,^7^,^8^,^9)^.

Mobility is closely related to other basic everyday activities, such as access to education, work, leisure, social interactions, and activities of daily living. Therefore, mobility issues can impact the quality of life, autonomy, and accessibility of visually impaired individuals^(4^,^5^,^6^,^7^,^8^; ^10)^.

Individuals with visual impairment and blindness commonly utilize the white cane to improve mobility and autonomy safely. The white cane is considered assistive technology. Assistive technologies are resources that enable or facilitate task execution by individuals with disabilities^(8^,^10)^. The white cane is currently the most commonly used assistive technology. It was developed in the United States to attenuate mobility problems common among visually impaired individuals. By its development, the first cane was longer and lighter than regular support canes. It was meant to be an extension of the indicator finger to provide a tactile-synesthetic perception of the space ahead, detecting the nature and conditions of the ground and the presence of obstacles, depressions, uphill and downhill, and reference points. This way, the cane could protect the inferior part of the body against collisions^(9)^.

There are currently two types of white cane tips: roller and regular. The roller is ideal for scanning the surface by movement, and the regular tip recognizes the surface type^(9^,^10^,^11^,^12^,^13).^

Although the long cane is considered the most effective and used assistive technology by individuals with visual impairment, it has some limitations, such as the short range of obstacle detection (less than two steps or at a distance equal to the cane length) and the inability to detect obstacles at head level^(11^,^12)^.

Due to these limitations, many efforts have been made to develop and commercialize products to supply the existing demand in the mobility field. Some examples are bracelets and other wearable devices, systems attachable to long canes, and electronic canes, all of them with the primary purpose of identifying or detecting the presence of obstacles in the user’s route and notifying them either by sound or vibration feedback (classified as mobility aids). Some provide location information through the Global Positioning System, which is classified as an aid to navigation^(11^,^12^,^14^,^15^,^16^,^17^,^18^,^19)^.

**Figure 1:**
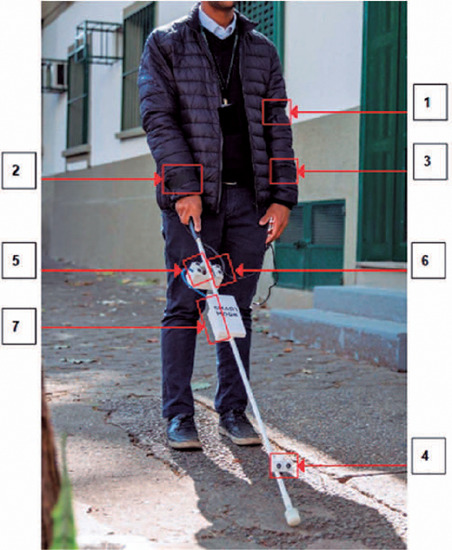
The wearable haptic interface attached to the electronic cane.

Source: Alex Reipert (Photographer; Universidade Federal de São Paulo).

Note: (1) Wristband A notifes the presence of obstacles on the ground. (2) Wristband B notifes the presence of obstacles higher to the right. (3) Wristband C notifes the presence of obstacles higher to the left. (4) Sensor A detects obstacles on the ground. (5) Sensor B detects obstacles higher to the right. (6) Sensor C detects obstacles higher to the left. (7) Portable power bank^(20)^.

Previously, at the Universidade Federal de São Paulo in Brazil, an electronic cane with a wearable haptic device was developed to assist in the mobility of individuals with visual impairment^(20^,^21)^. This cane notifies the user through the vibration of its wristbands when obstacles are detected within a distance of 1 m (vibration pattern keeps the same regardless of the distance to the obstacle-the system does not indicate the distance to the obstacle but notifies its presence). This system is composed of three hook and loop wristbands that contain three vibracall motors each, three ultrasonic sensors HC-SR04 for the detection and direction of obstacles, one microcontroller Arduino© board, one long cane with a roller tip, and a portable energy bank (Figure 1)^(20^,^21)^.

This study aimed to evaluate the perception and satisfaction of blind individuals regarding an electronic cane with a wearable haptic device.

## METHODS

This study was approved by the Universidade Federal de São Paulo Research Ethics Committee under number 0319/2019 (CAAE: 10425819.2.0000.5505) and followed the basic principles of the Declaration of Helsinki. All study participants agreed to participate by signing a consent form.

### Participants

Individuals were included in this study if they were legally blind, with a best corrected visual acuity of <20/400 (1.3 logMAR) in the best eye measured by the Early Treatment Diabetic Retinopathy Study table and/or visual field <10°, age ≥18 years, and previous experience with the long cane. Exclusion criteria were any cognitive, neurological, or motor deficit.

### Sociodemographic and blindness-related information

Sociographic and blindness-related information was initially acquired from participants. Etiology of vision loss, current use of a cane along with the type of cane, time of use, participation in the Navigation and Mobility course and duration of the course, use of other assistive technologies, which technologies, for how long, and if any orientation/training was done were asked of participants.

### Tests

Scenarios A and B were created with obstacles of different nature [cardboard, vinyl acetate (EVA), and aluminum] so that volunteers could travel through using their white cane and the prototype developed at the Universidade Federal de São Paulo. There was no difference between the scenarios; their only purpose was to prevent individuals from memorizing the route: both presented the same difficulty level as the same obstacles were used but only in opposite directions. A stratified randomization was used to define the scenario in which the individual would use their cane and the scenario in which they would use the prototype.

The participant was asked to perform the route of the defined scenario using his white cane, trying to avoid collision with the obstacles and using the time and speed necessary to complete the route. After finishing, the same participant was asked to perform the route of the other scenario, now using the prototype (described in the Introduction and references 20 and 21), based mainly on the vibration of wristbands of the prototype’s wearable system to avoid collision with obstacles. The number of collisions was counted by two people: one from the study team and the other, out. The time was marked by the same person from the study team using a conventional chronometer.

### Perception and satisfaction regarding the cane prototype

Participants gave feedback about the prototype’s general usefulness and functions, motor vibration, and the effectiveness of its sensors. In addition, participants were asked to compare their cane in use to the prototype in terms of usability (respecting the fact that one is already a consolidated product and the other an early prototype) and assessed stress and mental state while using both. This assessment was carried out through the application of a structured survey in an interview format.

### Data analysis

Data collected in this study were analyzed using Minitab® version 20.2 (Minitab, LLC, USA). The statistical model paired t-test and Wilcoxon’s nonparametric model were used to analyze the variables of the number of collisions and travel time between the prototype and the cane in use. The level of statistical significance was p≤0.05.

## RESULTS

Ten blind individuals participated in this study: 8 males and 2 females, ages between 23 and 43 years (mean 32.2 ± 7.13 years and median 32 years). Visual acuity values in both eyes ranged from 1.6 to 3 logMAR (mean 2.71 ± 0.44 logMAR and median 2.85 logMAR). Age at onset of blindness ranged from 1 to 30 years (mean 18.1 ± 9.46 years and median 19.5 years). The most common cause of blindness in this sample was retinitis pigmentosa (n=4, 40%).

### Sociodemographic and blindness-related information

Only 1 participant (10%) received government benefits from retirement due to disability. The average monthly income of most participants (n=5, 50%) ranged from 1 to 3 minimum wage (~209 USD/month). Regarding the level of education and professional performance, all participants at least completed high school; however, despite having some educational qualification, only 5 participants (50%) worked, whether formal or autonomous.

Regarding mobility, 8 participants (80%) reported that they leave home alone without problems. In contrast, 1 (10%) leaves home as long as he/she has company, and 1 (10%) does not leave the house because of ongoing treatment for depression. Only 1 participant (10%) lives alone.

Nine participants (90%) used a long/white cane with a roller tip, whereas 1 (10%) used a regular tip. Of the 10 participants, 9 declared they had participated in the Navigation and Mobility course, and the average duration of participation was 1.2 ± 0.63 years with a median of 1 year. The duration of cane use varied from 5 to 26 years, with an average of 12.4 ± 6.09 years and a median of 11 years.

Among the suggestions for improvements pointed out by participants about the cane currently in use, 5 participants (50%) pointed to obstacle detection, and four (40%) mentioned ergonomics.

### Tests

**Figure 2:**
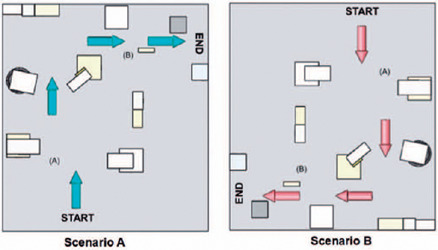
Drawings of scenarios A and B used to test the participant’s cane and the prototype.

Note: To the left, the scenario A, to the right, the scenario B. Squares and rectangles represent the cardboard and EVA boxes in both scenarios. Black circle, a hollow aluminum trash can. The colors of the obstacles are for aesthetic purposes only. (A) Detection of obstacles on the sides of the course was evaluated. (B) Detection of a frontal obstacle. Red arrows indicate the direction in which the test was performed.

Figure 2 displays the drawings of the scenarios utilized for tests with the participant’s cane and the prototype.

**Figure 3:**
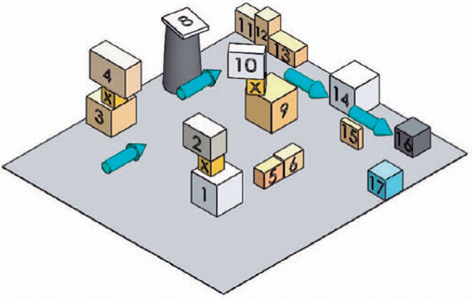
Three-dimensional representation of the obstacle distribution used in the tests conducted with the participant’s cane and the prototype.

Figure 3 displays a three-dimensional drawing of the obstacle distribution used in the tests conducted with the participant’s cane and the prototype.

Table 1 presents the dimensions of the boxes used as obstacles in the scenarios to validate the prototype.

**Table 1:** Dimensions of the boxes used as obstacles in the scenarios to validate the prototype

Obstacles	Height (cm)	Width (cm)	Length (cm)
1	49	46	46
2	66	56	29
3	42	45	45
4	57	37	29
5	25	19	35
6	28	20	35
8	6	49	39
9	48	47	47
10	56	37	20
11	24	48	23
12	38	35	20
13	24	48	23
14	46	45	45
15	31	32	9
16	32	32	32
17	32	32	32
X	18	24	23

As an exception, obstacle no. 7 was not a box but a trash can. Because its dimensions differed from those of a box, they are displayed here: diameter 94 cm, height 95 cm, radius #1 40 cm, and radius #2 50 cm. Three support boxes (X) were also used to set obstacles 2, 4, and 10 higher to better simulate aerial obstacles during the route.

Table 2 shows the data collected during the route completion with each cane. There was no statistically significant difference regarding the number of collisions and travel time in the tests, regardless of the cane used.

**Table 2:** Number of collisions and travel time with the participant’s cane and the prototype [average ± SD (median)]

	Participant’s cane	Electronic cane prototype	p
Collisions between cane and ground obstacles	15.2 ± 8.36 (14)	10.3 ± 10.1 (5.5)	0.081
Collisions between cane and aerial obstacles	0.4 ± 0.97 (0)	0.4 ± 0.70 (0)	1.000
Collisions between participants and obstacles (ground and aerial)	1.6 ± 1.95 (0.5)	2.0 ± 1.82 (1)	0.534
Time (s)	61.4 ± 66.6 (44)	75.1 ± 78.4 (51)	0.634

### Perception and satisfaction regarding the cane prototype

All participants declared that the wristband motors worked without problems. Five participants (50%) considered the prototype feedback better than their cane’s. Regarding the intensity of the wristband vibration, 7 participants (70%) considered it adequate. The other 3 participants (30%) considered the vibration slightly inconvenient.

Nine participants (90%) felt safe using the prototype. As for the degree of difficulty in testing the prototype, only 2 participants (20%) considered it moderate, whereas the other 8 (80%) reported slight or no difficulty using the prototype.

All participants considered the electronic cane prototype useful, and 7 (70%) suggested changes in the prototype that were reducing the number of sensors (20%), improving the detection of higher obstacles (20%), detection range (10%), type of feedback (10%), and motor vibration intensity (10%).

The scores on the individual’s cane ranged from 6 to 10 (mean 8 ± 1.49 and median 8), and scores given to the prototype ranged from 5 to 10 (mean 7.9 ± 1.43 and median 8). The differences between scores given to the individual’s cane and the prototype were considered insignificant (p=0.80).

Seven participants (70%) mentioned the novelty/little experience in using the prototype more frequently as a challenge to the performance of the tests, followed by the prototype wearable system cables.

## DISCUSSION

The general evaluation of the prototype made by participants was satisfactory, as its hardware worked flawlessly, its concept was considered helpful for the mobility aid proposal, and the average score attributed to it had little variation about the individuals’ cane, not showing statistical significance.

Although the prototype’s purpose is not to prevent collisions, informing its user of obstacles’ presence, there was a tendency for fewer collisions with ground obstacles when the prototype was used, indicating that the prototype can solve a vital mobility challenge in the visually impaired population-the obstacle detection in their route, in agreement with previous literature^(22)^.

The detection precision of the ultrasonic sensor of the prototype depends on the obstacle’s nature. However, during the tests, there were no noticeable differences between obstacle detection regardless of its nature^(22)^.

During the route taken with the prototype and the individual’s cane, there were fewer collisions with terrestrial obstacles while using the prototype. For aerial obstacles, this difference was not observed. The number of collisions between the participant and both types of obstacles and the total travel time were less while using the individual’s cane. Similar results were obtained in another study comparing a prototype of an electronic cane to a white cane. It is worth mentioning that when detecting obstacles in more than one direction (i.e., upper and lower or upper right and upper left), all wristbands involved in each notification are activated^(23)^.

It is also important to clarify that the purpose of the tests with canes was to understand the prototype functionality, not its effectiveness. For this reason, the order of the cane to be used was not randomized. For the same reason, usability and ergonomics questionnaires, such as System Usability Score and Nasa TLX, are useful for assessing technologies in the “product phase”. The cane assessed in this study was in a prototype phase, a proof of concept.

Ergonomic improvements to the prototype are necessary to meet the participant’s suggestions, including the handle, its handhold, and the material of the cane. An increase in the number of individuals for the tests would also aid in understanding the best alternative for detection of obstacles, mostly suggested by participants in terms of changes in their current cane in use.

Data collection was performed during the coronavirus disease 2019 (COVID-19) period. For this reason, it was not possible to enroll more participants. No more than one test for each situation was performed to decrease the exposure and risk for each participant. Moreover, all safety measures to avoid COVID-19 were taken. For this same reason, enrollment preference was given to younger ones with no comorbidities and residents in the city of the institution where the study was performed.

Some participants suggested that even higher obstacles should be used so that the upper sensors can detect this type of obstacle. This may be why the number of collisions with aerial obstacles was much smaller than those with terrestrial obstacles. Maybe the obstacles were not as high as they should be for the sensors to detect. In this case, in addition to improving the arrangement and type of obstacles, it was necessary to calculate and adjust the ideal position for the prototype sensors to ensure that air and ground obstacles are detected by their respective sensors.

All participants preferred their canes to move on the proposed routes during the tests. Of these, 5 participants (50%) mentioned the lack of experience in using the prototype as one of the reasons for this choice. A validation study of an electronic cane prototype with 20 visually impaired individuals suggested the implementation of prior training so that the individual acquires substantial experience using the prototype because they already have extensive experience using their cane^(23)^. Therefore, in future studies, including a prior training period with the prototype would improve performance in its use and refine the test results.

In this study, blind individuals who participated understood and were satisfied with the prototype of an electronic cane developed to help mobility. Future studies must be conducted to verify the items pointed out by participants of this study, taking into account a larger population of legally blind individuals.
